# Transcriptome Analysis Revealed the Mechanism by Which Exogenous ABA Increases Anthocyanins in Blueberry Fruit During Veraison

**DOI:** 10.3389/fpls.2021.758215

**Published:** 2021-11-11

**Authors:** Tianyu Han, Wenlong Wu, Weilin Li

**Affiliations:** ^1^Co-Innovation Center for Sustainable Forestry in Southern China, Forestry College, Nanjing Forestry University, Nanjing, China; ^2^Institute of Botany, Jiangsu Province and Chinese Academy of Sciences, Nanjing, China

**Keywords:** blueberry, anthocyanins, transcriptome, ABA, *VcMYBA*

## Abstract

Blueberry (*Vaccinium* spp.) is a popular healthy fruit worldwide. The health value of blueberry is mainly because the fruit is rich in anthocyanins, which have a strong antioxidant capacity. However, because blueberry is a non-model plant, little is known about the structural and regulatory genes involved in anthocyanin synthesis in blueberries. Previous studies have found that spraying 1,000 mg/L abscisic acid at the late green stage of “Jersey” highbush blueberry fruits can increase the content of anthocyanins. In this experiment, the previous results were verified in “Brightwell” rabbiteye blueberry fruits. Based on the previous results, the anthocyanin accumulation process in blueberry can be divided into six stages from the late green stage to the mature stage, and the transcriptome was used to systematically analyze the blueberry anthocyanin synthesis process. Combined with data from previous studies on important transcription factors regulating anthocyanin synthesis in plants, phylogenetic trees were constructed to explore the key transcription factors during blueberry fruit ripening. The results showed that ABA increased the anthocyanin content of blueberry fruits during veraison. All structural genes and transcription factors (MYB, bHLH, and WD40) involved in the anthocyanin pathway were identified, and their spatiotemporal expression patterns were analyzed. The expression of *CHS*, *CHI*, *DFR*, and *LDOX/ANS* in ABA-treated fruits was higher in the last two stages of maturity, which was consistent with the change in the anthocyanin contents in fruits. In general, six MYB transcription factors, one bHLH transcription factor and four WD40 transcription factors were found to change significantly under treatment during fruit ripening. Among them, *VcMYBA* plays a major role in the regulation of anthocyanin synthesis in ABA signaling. This result preliminarily explained the mechanism by which ABA increases the anthocyanin content and improves the efficiency of the industrial use of blueberry anthocyanins.

## Introduction

Blueberry belongs to the genus *Vaccinium*, and the main cultivated species are northern highbush blueberry plants (*Vaccinium corymbosum* L.), southern highbush blueberry (primarily *V. corymbosum* L.), lowbush blueberry (*V. angustifolium* Aiton), and rabbiteye blueberry (*V. ashei* Reade) (Leisner et al., 2017). Blueberry fruit is one of the most popular healthy fruits worldwide. This popularity is mainly because blueberries contain a variety of phytonutrients, the most representative of which are anthocyanins (Borges et al., 2010). With the in-depth study of blueberry anthocyanins, a growing number of clinical and animal experiments have proven that blueberry anthocyanins can effectively alleviate obesity (Prior et al., 2010) and cardiovascular disease (Zhu et al., 2013) and prevent type 2 diabetes (Burton-Freeman et al., 2019) and cancer (Faria et al., 2010). Therefore, the content and variety of anthocyanins are important characteristics of blueberry fruit. This study systematically explored the key transcription factors in anthocyanin synthesis and laid a foundation for future molecular breeding.

The structural genes of the anthocyanin pathway in plants are well understood, and the important functions of *CHS* (Schijlen et al., 2007), *CHI* (Guo et al., 2015), *DFR* (Katsu et al., 2017), *ANS* (Zhang et al., 2015), and *UFGT* (Boss et al., 1996) have also been verified. At present, three kinds of transcription factors, MYB, bHLH, and WD40, have been found to play a major regulatory role in the anthocyanin pathway (Dubos et al., 2010). They form the MBW complex and directly regulate the expression of structural genes, among which MYB transcription factors play a major role (Gonzalez et al., 2008). In *Arabidopsis thaliana*, the expression levels of *AtMYB75*, *AtMYB90* (Borevitz et al., 2000), *AtMYB113* and *AtMYB114* (Gonzalez et al., 2008) were positively correlated with changes in the anthocyanin content. Similarly, *GL3* and *EGL3* (Zhang et al., 2003) of the bHLH family identified in *Arabidopsis thaliana* also showed a positive correlation. The loss of *AtTTG1* in the WD40 family can affect the expression of DFR and other anthocyanin synthesis genes (Gonzalez et al., 2008). However, there are few studies on the structural genes and transcription factors regulating anthocyanin synthesis in blueberry. Only one MYB transcription factor, *VcMYBA*, has been found to increase anthocyanin synthesis by activating the promoter of *DFR* (Plunkett et al., 2018).

Exogenous ABA can increase the anthocyanin content of non-climacteric fruits such as strawberry (Jia et al., 2011; Li et al., 2011) and grape (Sandhu et al., 2011; Yamamoto et al., 2015). Exogenous ABA was also effective for non-climacteric fruit blueberry, and the northern highbush blueberries were found to have accelerated coloration and increased anthocyanin content 12 days after treatment with 1,000 mg/L exogenous ABA application (Oh et al., 2018). Therefore, the discovery that ABA treatment increased the total anthocyanin content of blueberry fruits can be used to study the mechanism of anthocyanin synthesis. With the development of high-throughput sequencing, the transcriptome has been widely used to study metabolic pathways and key genes. The genome of highbush blueberry (*V. corymbosum*) was assembled, which could provide a good reference for studies on its transcriptome (Colle et al., 2019). The mechanism of exogenous ABA application on grape berry ripening at 22 and 44 h was systematically illustrated by RNA-seq (Pilati et al., 2017). Similarly, the transcriptome can be used to systematically study the synthesis mechanism and explore the key regulatory genes in blueberry.

In this study, blueberry fruit was divided into six stages from late green to mature. We systematically analyzed the effect of ABA on whole transcripts during fruit ripening, especially those involved in the anthocyanin pathway. Combined with data from previous studies on anthocyanin biosynthesis in plants, key transcription factors involved in anthocyanin biosynthesis were identified, and their expression patterns were analyzed. Among the key transcription factors, the expression of *VcMYBA* was consistent with the increase in anthocyanins and ABA-responsive elements were found in the promoter of *VcMYBA*, suggesting that *VcMYBA* may play a role in ABA pathway. Transient silencing experiments showed that *VcMYBA* played an important role in anthocyanin synthesis.

## Materials and Methods

### Plant Materials and ABA Treatments

Four years old rabbiteye blueberry “Brightwell” were grown in experimental base of Baima district, Nanjing city, China. Blueberries of same size and growing condition were selected to do the treatment. Each treatment was arranged in a randomized complete block design with 6 replications and each block replication contains 6 shrubs.

The (+)-Abscisic Acid (Purity 95%, Coolaber company, China) was dissolved in double distilled H_2_O containing 5% (v/v) ethanol and 0.1% Tween 80. When most of blueberries fruits were in a growing stage of “green mature” and the fruit at the top of the branch has just begun to turn red, 0, 500, and 1,000 mg/L ABA solutions were sprayed on fruit clusters. Tiny sprayer were used to spray the ABA solutions on the peels untill the peels are wetted. The fruit is guaranteed to be sprayed 2–3 times on all sides. Although the mock treatment was performed on blueberry shrubs, leaves, and branches were carefully avoided.

In different development stage of fruits, 30 fruits were immediately frozen in liquid nitrogen and stored at −80°C for later experiments.

### Physiological Characterization

#### The Color of Fruit Peel

Fruit peel color was measured by colorimeter (Ci64, X-Rite, United States) and shown by the International Commission on Illumination a^∗^ and b^∗^ color space co-ordinates (Hunter and Harold, 1987). The a^∗^ value is negative for green and positive for red and the b^∗^ value is negative for blue and positive for yellow, both of the values range from −100 to 100. Due to the coloration of rabbiteye fruits starts from top to bottom, the top, side and bottom of fruit peel were separately measured in the same stage.

#### The Total Anthocyanins Content

The total anthocyanins content were determined by the double pH differential method (Pantelidis et al., 2007): absorbance of the extract was measured at 510 and 700 nm in buffers at pH 1.0 (hydrochloric acid–potassium chloride, 0.2 M) and 4.5 (acetate acid–sodium acetate, 0.2 M). Total anthocyanins content was calculated using a molar extinction coefficient of 29,600 (cyanidin-3-glucoside) and absorbance of A = [(A510 − A700)_*pH* 1_._0_ −(A510 − A700)_*pH* 4_._5_].

#### Fruit Hardness

The fruit hardness were determined by Fruit hardness tester (Catno.9300, Takemura Electric Works Co., Japan). The cone type tip was used and the tip was perpendicularly applied on the side surface of blueberry fruits. The value was measured at the moment of tip intrusion to the surface.

#### Brix

The Brix were determined by saccharometer (PAL-1, Atago Co., Japan). The juice were left on the prim for 20 s to do the measurement.

### Transcriptome Analysis

#### RNA Preparation and Sequencing

Total RNA was extracted from whole fruits using Trizol reagent (Invitrogen, Carlsbad, CA). The RNA integrity was analyzed on agarose gel. RNA concentration and integrity were measured by Qubit^®^ RNA Assay Kit in Qubit^®^ 2.0 Flurometer (Life Technologies, CA, United States) and RNA Nano 6000 Assay Kit of the Bioanalyzer 2100 system, respectively. Sequencing libraries were generated using NEBNext^®^ Ultra^TM^ RNA Library Prep Kit for Illumina^®^ (NEB, United States) following manufacturer’s recommendations. Then, the libraries were sequenced on an Illumina Hiseq platform and 150 bp paired-end reads were generated.

#### Reads Mapping to the Reference Genome

Clean reads were obtained by in-house Perl scripts that remove reads containing adapter, reads containing ploy-N and low quality reads from raw reads. Reference genome and gene model annotation files of *V. corymbosum* (version 1.0) were downloaded from Giga science^1^ (Colle et al., 2019). Paired-end clean reads were aligned to the reference genome using STAR (v2.5.1b) by the method of Maximal Mappable Prefix. The function of the transcript was annotated by UniProt database.^2^

#### Differential Expression Analysis

HTSeq v0.6.0 was used to count the reads numbers mapped to each gene and FPKM (fragments per kilobase per million reads) was calculated based on the length of the gene and reads count mapped to this gene. Then, differential expression analysis of two groups was performed using the DESeq2 R package (1.10.1). Genes with *P*-value using Benjamini and Hochberg’s approach were assigned as differentially expressed. Enrichment analysis of differentially expressed genes of physiological processes was carried out by KEGG^3^ and GO (clusterProfiler R package).

#### Construction of Phylogenetic Trees

The maximum likelihood phylogenetic trees was constructed by IQ-tree (Nguyen et al., 2015). The optimal alternative model is selected after calculation. The bootstrap value is 1,000. The following sequence was downloaded from GenBank accessions: **MYB:** *AtMYB75*(NC_003070.9), *AtMYB113*(NC_003070.9), *AtMYB90* (NC_003070.9), *AtMYB114*(NC_003070.9), *PhAN2*(AAF66727.1), *PhPHZ*(ADQ00388.1), *PhDPL*(ADQ00393.1), *VvMYBA1* (BAD18977), *VvMYBA2*(BAD18978), *MdMYB10*(ACQ45201), *MdMYBA*(NC_041797.1), *VcMYBA*(MH105054), *AcMYB10*(PSS35990), *AcMYB110*(AHY00342), *AtMYB4*(At4g38620), *PhM YB4*(ADX33331.1), *PhMYB27*(AHX24372), *VvMYBC2-L2*(ACX50288.2), *VuMYBC2*(AKR80571), *VvMYBC2-L1*(AFX64995.1), *AtMYB12*(ABB03913), *SlMYB12*(ACB46530.1), *VvMYBF1*(ACV81697), *AtMYB123*(CAC40021), *VvMYBPA2*(ACK56131.1), *Vc MYB17*(ALP43798.1), *MdMYB11*(NC_041797.1), *MdMYB9* (NC_041796.1), *VuMYBPA1*(AKC94840.1), *VvMYBPA1*(CAJ90831.1), *AtMYB5*(NP_187963.1), *VvMYB5b*(AAX51291), *AtM YB56*(AT5G17800), *MdMYB1*(NC_041789.1). **bHLH:** *AtEGL3* (AT1G63650), *AtGL3*(AT1G11130), *AmDELILA*(Uniprot:Q38736), *PhJAF13*(Uniprot:O64908), *AtTT8*(AT4G09820), *MdbH LH3*(ADL36597.1), *PhAN1*(FJ227329.1), *VvbHLH1*(Uniprot:A0A438KI27), *MdBHLH33*(EI011581.1). **WD40**:*AtTTG1*(AT5G24520), *MdTTG1*(GU173814.1), *PhAN11*(Uniprot:O24514), *Vv WDR1*(Uniprot:Q19N39), *VvWDR2*(Uniprot:Q19N38).

#### Quantitative Real-Time PCR Analysis

Eleven transcription factors with altered expression levels were selected for qRT-PCR verification. The specific primers for 11 genes are shown in Supplementary Table 4. Expression level is relative to the housekeeping gene *Actin* of highbush blueberry (Zifkin et al., 2012). qPCR was conducted using the TB Green^®^ Fast qPCR Mix (Takara, Japan) according to manufacturer’s requirements. The 2^–ΔΔCt^ method was used to calculate the relative gene expression (Livak and Schmittgen, 2001). The experiment carried out three biological repetitions.

#### Virus-Induced Gene Silencing in Blueberry Fruit

In order to identify the function of four copies of *VcMYBA*, transient silencing was performed by the tobacco rattle virus based VIGS technology. Because the other four sequences are similar and the three coding sequences are shifted, they cannot be translated into proteins. Therefore, interference sequences are designed in exon region and intron region, respectively. The length of the two sequences was about 330 bp, which was amplified by PCR with specific primers (Supplementary Table 6). The PCR products were cloned into the pTRV2 vector to produce TRV: VcMYBA-exon and TRV: VcMYBA-intron constructs. The *Agrobacterium tumefaciens*, strain GV3101, was used in transforming the recombinant plasmids pTRV1, pTRV2. Infection solution formula: 0.01 mM MgCl_2_, 0.01 mM MES, 200 μM AS, 0.6OD Agrobacterium solution (pTRV1 and pTRV2 mixed in equal proportion). Slowly inject the infection solution to the side of the fruit. Observe the fruit after 10 days.

#### Analysis of Anthocyanin Changes in Fruits by UPLC/MS

UPLC-MS/MS analysis was performed to evaluate anthocyanin changes in transiently silenced fruits. The standards of petunidin, cyanidin, delphinidin, pelargonidin, and peonidin (Coolaber company, China) were used to make the standard curve. 200 mg fruit powder was added 5 ml of extraction solution (Absolute ethanol: H_2_O: HCL = 2:1:1) and ultrasonic extraction in water bath at 4°C for 0.5 h. Then, the samples were hydrolyzed in boiling water bath for 60 min. After that, the samples were taken out and cooled, and the volume of the samples was fixed to 5 ml. The samples were left standing and the supernatant was transferred to the fresh tubes. Finally, the supernatants were filtered through 0.22 μM filter membrane and loaded on instrument for analysis. LC-MS/MS analyses were performed using a LC-30AD system (Shimadzu) coupled with an Qtrap6500 mass spectrometer (AB SCIEX). Samples were injected onto an Agilent Zorbax sb-c18 column (150 × 4.6 mm, 1.9 μm) using a 16-min linear gradient at a flow rate of 1 m/min. The eluents were eluent A (0.1% formic acid) and eluent B (0.1% acetonitrile). The solvent gradient was set as follows: 18% B, 0 min; 20% B, 4 min; 35% B, 7 min; 95% B, 8 min; 95% B, 12 min; 18% B, 12.1 min; 18% B, 0 min, 16 min. Mass spectrometer was operated in ESI positive polarity mode with spray voltage of 5.5 kV, capillary temperature of 550°C, atomization gas pressure of 50 psi and auxiliary air pressure of 60 psi.

## Results

### Fruit Coloration, Total Anthocyanin Content, and Fruit Quality

**FIGURE 1:**
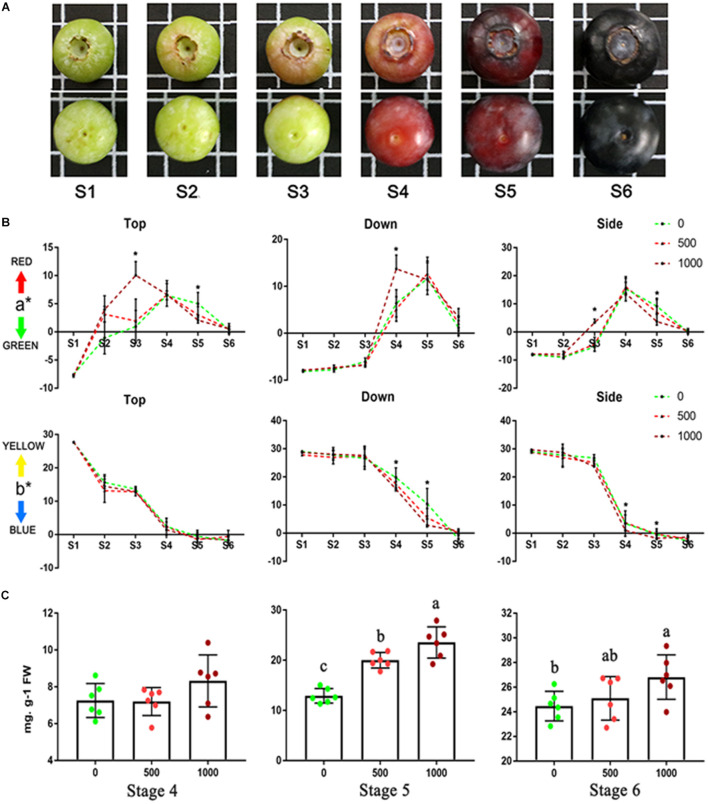
Ripening progress of blueberry (cv “Brightwell”) and coloration and anthocyanins content of fruits under 0, 500, and 1,000 mg/L ABA treatment. **(A)** The ripening progress is divided into six stages. **(B)** The color of top, side and bottom of fruits from S1 to S6. **(C)** Total anthocyanins content from S4 to S6. Different letters and asterisk indicate statistical significance (*P* < 0.05) as determined by a one-way ANOVA test (Duncan’s multiple range).

The developmental stage of cv “Brightwell” from ripening initiation to maturity was divided into six stages (Figure 1A) based on fruit color (Stage 1, the whole fruit was green; Stage 2, the top turned red; Stage 3, the side turned red; Stage 4, the whole fruit was red; Stage 5, the whole fruit turned purple; and Stage 6, the whole fruit was purple) and size (Supplementary Table 1). Application of 1,000 mg/L ABA resulted in a significant acceleration of fruit coloration (Figure 1B). From S3 to S4, the a^∗^ value suggests that the top, side and bottom of fruit from the 1,000 mg/L ABA group turned red earlier than those from the 0 to 500 mg/L ABA groups. At S5, the top and bottom of the fruits turned green earlier. From S5 to S6, the b^∗^ value suggests that the side and bottom of the fruit from the 1,000 mg/L ABA group turned blue earlier than those from the 0 to 500 mg/L ABA groups. From the a^∗^ and b^∗^ values, there were no notable differences among the three groups at S6. Anthocyanin accumulation was consistent with the change in fruit coloration (Figure 1C). At S1–S3, almost no anthocyanins were detected. At S4, although the average anthocyanin content of the 1,000 mg/L treatment was higher than that of the control group, there was no statistically significant difference. At S5 and S6, the total anthocyanin content of the 1,000 mg/L ABA treatment group was obviously higher than that of the 0 and 500 mg/L treatment groups. From S5 to S6, the anthocyanin content of the 0 mg/L treatment group nearly doubled but was still lower than that in the 1,000 mg/L group. Application of 1,000 mg/L ABA also changed the fruit quality (Supplementary Figure 1). Compared to 0 and 500 mg/L ABA treatment, fruits hardness of 1,000 mg/L ABA treatment declined more sharply from S4 and become softer in S6 (Supplementary Figure 1A). Consist with fruit hardness, fruits of 1,000 mg/L ABA treatment contains more soluble solid in S6 (Supplementary Figure 1B). Application of ABA did not significantly change the fruit size, which was within the range of Supplementary Table 1. Therefore, blueberry fruits treated with 0 and 1,000 mg/L ABA were selected for transcriptome analysis.

### Assembly and Annotation of the Transcriptome

The fruits in Stages 1–6 with 0 and 1,000 mg/L ABA treatment were further subjected to transcriptome analysis. The raw bases of each sample varied from 7.04 G to 10.91 G with a 0.03% error ratio and 46% GC content. In total, 2218553864 clean reads and 2176923110 raw reads were obtained (Supplementary Table 2). The raw reads of 12 samples with three biological repeats were uploaded to the National Center for Biotechnology Information (NCBI) Sequence Read Archive (SRA) database (all samples and corresponding explanations can be found in PRJNA664011). For each sample, 87.43–89.19% of the reads were mapped to the highbush blueberry genome. Approximately 87% of reads were mapped to exonic regions, 7% of reads were mapped to intergenic regions, and 6% of reads were mapped to intronic regions. The clean reads were assembled into 128,559 unigenes. A total of 88,532 unigenes were annotated by the Swiss-Prot database, accounting for 68.86% of the total (Supplementary Table 3).

### Profiling of the Transcriptome

**FIGURE 2:**
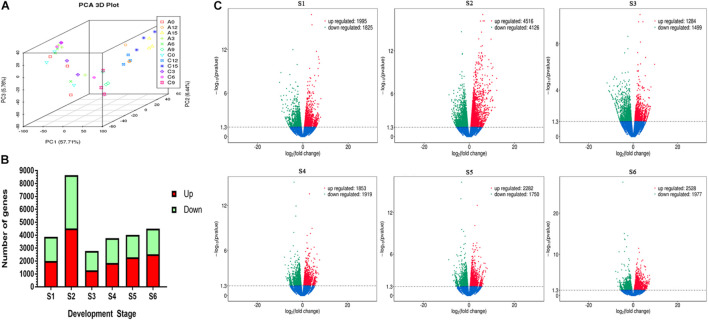
Profiling of transcripts difference between the two groups in six stages. **(A)** Principal component analysis of each sample. A0-15 represents 1,000 mg/L ABA treatment S1–S6, C0-15 represents S1–S6 of control group **(B)** The total number of transcripts significantly up-regulated and down regulated in development stages. **(C)** Volcano maps show overall differences between treatments.

Principal component analysis (PCA) was used to analyze the overall variation in transcripts among 32 samples. The 3D plot shows that the three repetitions are well clustered together, and the differences between the sample points are obvious (Figure 2A). The FPKM value of genes in the ABA-treated group was compared with that in the control group. A Log_2_(fold change) value greater than 1.3 or less than −1.3 indicates upregulation and downregulation of gene expression, respectively. At S2, the number of genes with significant changes was approximately twice as high as that of the control. The number of differentially expressed genes decreased sharply at S3 and then gradually increased at S4–S6 (Figure 2B). Volcano maps were used to represent the overall changes in gene expression between two groups (Figure 2C). The results showed that the number of genes with drastic changes in expression decreased continuously from S1 to S6.

**FIGURE 3:**
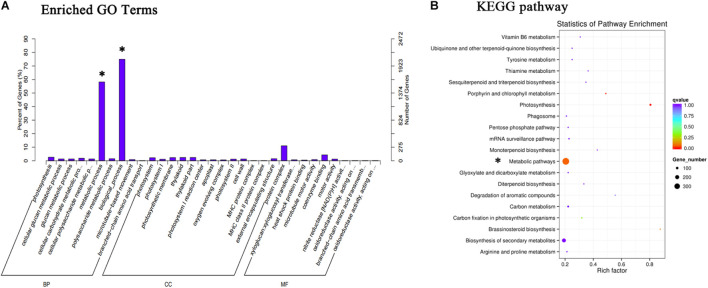
GO and KEGG were used to analyze the pathway of significant changes in S4. **(A)** Different expression genes were annotated and enriched with the GO database. **(B)** Metabolic pathways with different changes annotated by KEGG database. Asterisk marks changes in metabolic pathways.

The total amount of anthocyanins in the ABA treatment group was higher than that in the control group from S4, so the transcripts of fruits at S4 were functionally categorized and analyzed using the GO and KEGG databases (Figure 3). The results showed that the anthocyanin synthesis pathway had changed significantly.

### Structural Genes Involved in Anthocyanin Synthesis

**FIGURE 4:**
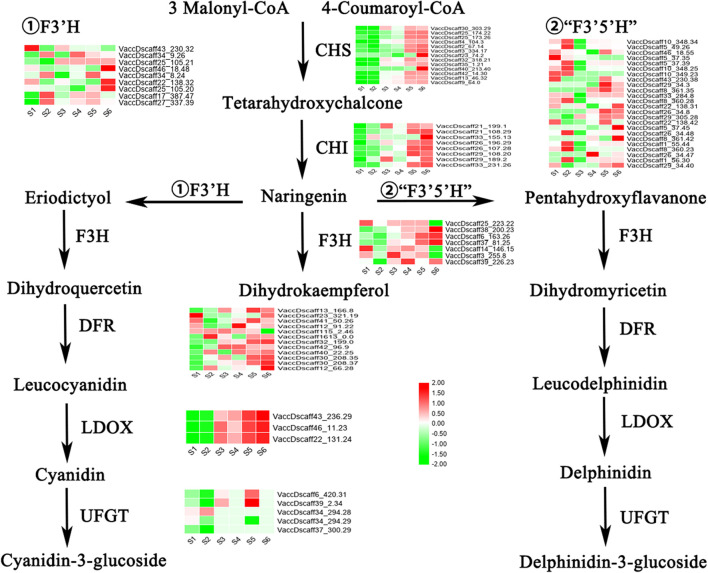
Expression changes of key structural enzymes in anthocyanin pathway during S1–S6 after ABA treatment. Red represents up regulation of gene expression, while green represents down regulation of gene expression. The expression levels (FPKM) corresponding to the different colors ranges from –2 to 2.

The transcripts of all key enzymes involved in the anthocyanin pathway were analyzed (Figure 4). Green indicates that the expression of transcripts in the ABA-treated group was lower than that in the control group, while red indicates the opposite. In the whole process, the transcripts of CHS and CHI, two important enzymes upstream, all showed consistent downregulation at S1 and S2 and increased at S5 and S6. Another key enzyme, LDOX/ANS, showed a similar expression pattern and was highly expressed at S3 and S4. The transcripts of the three enzymes F3H, F3′H and F3′5′H, which are found on different branches, showed different expression patterns across the six stages. Similarly, the expression of UFGT transcripts was inconsistent. Although the transcripts of DFR differed to some extent across the six stages, they were generally highly expressed after S3. In general, the transcript expression patterns of CHS, CHI, DFR, and LDOX were consistent with the changes in the anthocyanin content in the fruits.

### Identification of Key Transcription Factors Involved in Anthocyanin Synthesis

**FIGURE 5:**
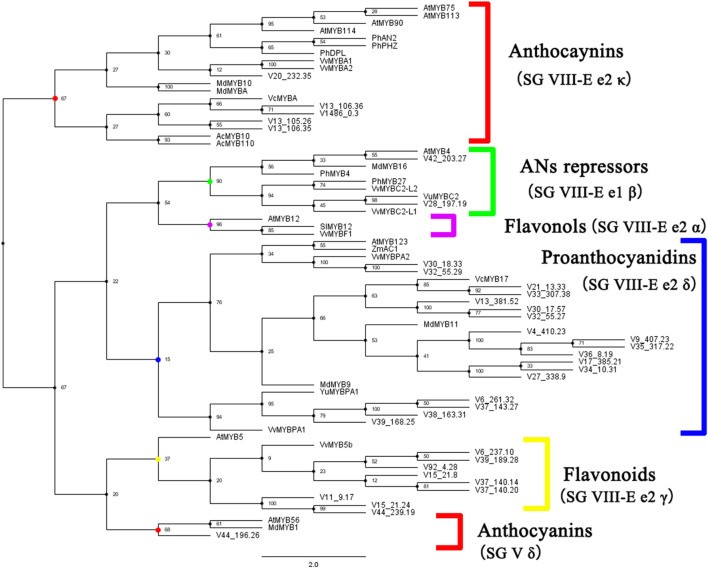
Identification of known MYB transcription factors regulating anthocyanin synthesis in blueberry. Phylogenetic tree of MYB transcription factors was constructed in whole nucleic acid sequences using maximum likelihood tree with 1,000 bootstrap in IQ-tree. The optimal alternative model was selected after calculation by IQ-tree.

The expression pattern of several key structural genes was the same, which indicates that the expression of transcription factors may affect the expression of downstream genes. Published transcription factors from different species were used to construct a phylogenetic tree with homologous transcription factors in blueberry. According to the classification of MYB transcription factors (Jiang and Rao, 2020) and their functions, MYB transcription factors involved in anthocyanin synthesis in blueberry were identified (Figure 5). Five blueberry MYB transcripts belong to the branch SG VIII-e2κ, and the expression of MYB transcription factors on this branch has been proven to promote anthocyanin synthesis. A blueberry transcript belonging to the SG V δ branch that promotes anthocyanin synthesis was also identified. For the negatively regulated SG VIII-E e1β branch, two transcripts were identified. For bHLH and WD40 transcription factors involved in anthocyanin regulation, four homologous genes were identified (Supplementary Figures 2, 3).

### Spatiotemporal Expression Analysis of Key Transcription Factors

**FIGURE 6:**
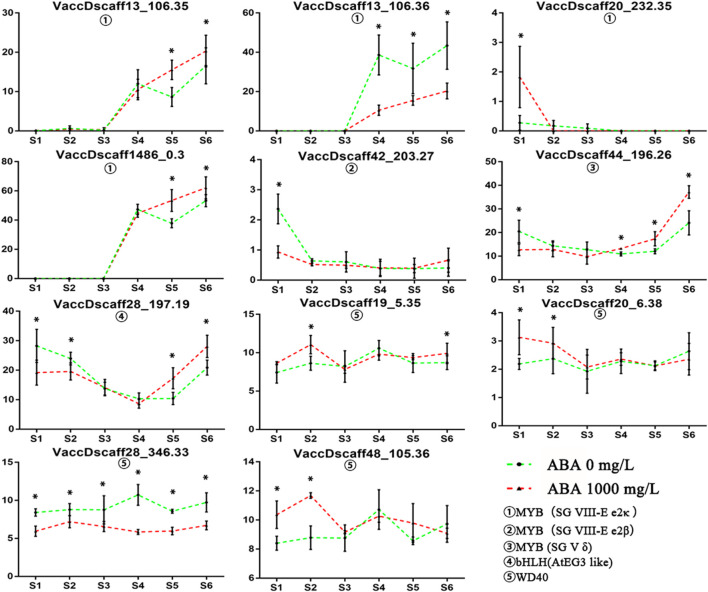
MYB, bHLH, and WD40 transcription factors with different expression in S1–S6. FPKM is used to represent the level of gene expression. Asterisk indicates statistical significance (*P* < 0.05) as determined by a one-way ANOVA test (Duncan’s multiple range). Values represent the means ± SD of three replicates.

Among the identified transcription factors, six MYB transcription factors, one bHLH transcription factor and four WD40 transcription factors showed significant differences in specific stages (Figure 6). The expression pattern of these transcription factors was verified by qRT-PCR (Supplementary Figure 4). The expression of two MYB transcription factors on the SG VIII-e2κ branch was positively correlated with anthocyanin content. At the same time, both transcripts are homologous genes of *VcMYBA* that have been proved to regulate anthocyanin synthesis. Two homologous negatively regulated MYB transcription factors showed upregulated and downregulated differential expression at S1. The transcript belonging to the SG V δ branch showed a high increase from S4 to S6, which was earlier than the change in the anthocyanin content. The bHLH transcript homologous to *AtGL3* was downregulated at S1–S2 but highly expressed at S4–S5. There was no correlation between the expression patterns of the four WD40 transcripts. The results showed that transcripts belonging to the SG VIII-e2κ branch and homologous to *VcMYBA* were most likely related to the increase in anthocyanins. Through promoter element analysis, 2-4 ABREs (ABA-responsive elements) was found to appear in the promoter region of four copies of *VcMYBA* (Supplementary Table 5). This shows that *VcMYBA* responds in the ABA pathway and may therefore be associated with the changes of *PYL* (Encodes regulatory components of ABA receptor), *SnRK2* (protein kinases involved in ABA signaling), and *ABF* (bZIP transcription factor with specificity for ABRE) gene families (Supplementary Figure 6). After ABA treatment, seven genes of *ABF* family were up-regulated in S1–S6, but the up-regulation degree decreased gradually. In S4, the expression of four ABF genes in the treatment group was 1.27–1.5 times higher than that in the control group. *PYL* and *SnRK2* gene families did not show an obvious overall change trend, but one gene in *PYL* family changed 1.79 times in S3 and one gene in *SnRK2* family changed 1.59 times in S4. From the expression of these genes, *VcMYBA* has a positive correlation with their expression.

### Transient Silencing of the Expression of Four Copies of *VcMYBA*

**FIGURE 7:**
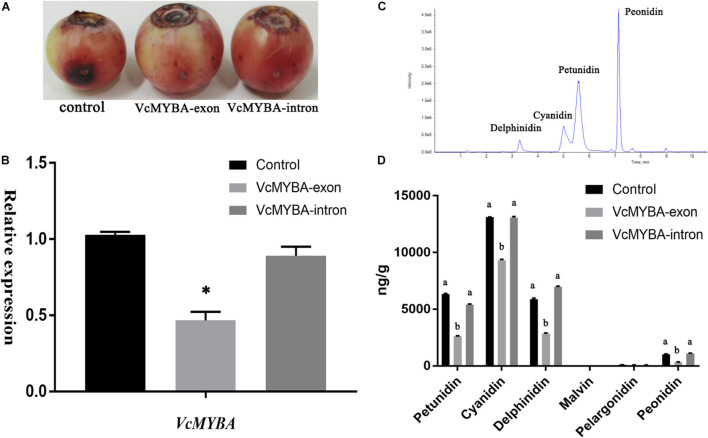
Virus-induced gene silencing of *VcMYBA* in blueberry fruit. **(A)** Transgenic fruits 10 days after VIGS treatment. **(B)** qRT-PCR analysis of *VcMYBA* expression in treated fruits. **(C)** UPLC analysis of anthocyanins. **(D)** Contents of six anthocyanins in treated fruits. Values represent the means ± SD of three replicates. Different letters and asterisk indicates statistical significance (*P* < 0.05) as determined by a one-way ANOVA test (Duncan’s multiple range).

Through the genome, a total of five copies of *VcMYBA* were identified. One copy had transposon insertion, two copies had frameshift and could not be translated into protein, one copy of TCA base sequence was mutated into the termination codon TGA and only one copy could be fully translated into protein (Supplementary Figure 5). Because of their high sequence similarity, transient silencing was designed in exon and intron regions, respectively. The results showed that in the control group, the anthocyanin synthesis in the injection wound area increased, and the anthocyanin synthesis in the fruit treated with TRV: *VcMYBA*-exon decreased, while the anthocyanin accumulation in the wound treated with TRV: *VcMYBA*-intron was not obvious, and the anthocyanin synthesis in the fruit did not decrease significantly (Figure 7A). The expression of *VcMYBA* in TRV:*VcMYBA*-exon group was also significantly lower than that in control group and TRV:*VcMYBA*-intron group (Figure 7B). The contents of six main anthocyanins were detected by UPLC-MS/MS (Figure 7C). The contents of petunidin, cyanidin, delphinidin, and peonidin in TRV: *VcMYBA*-exon group were significantly lower than those in the other two groups (Figure 7D). There was no significant change in anthocyanin content in TRV:*VcMYBA*-intron compared with the control group.

## Discussion

### Effect of ABA on Blueberry Fruit

Many studies have reported that exogenous ABA could increase the anthocyanin content in non-climacteric fruits (Jia et al., 2011; Sandhu et al., 2011). The anthocyanin content of plants will also increase under many abiotic stresses, such as drought (Nakabayashi et al., 2014), low temperature (Li et al., 2017), and nitrogen deficiency (Zhang et al., 2017). The response of plants to these stresses is also related to the ABA signaling pathway. Previous studies in blueberry only focused on the 12 days after ABA application at the late green fruit stage (Oh et al., 2018). The results showed that the content of anthocyanins increased and the color of blueberry fruits changed faster after 1,000 mg/L ABA treatment, which was consistent with the results of other non-climacteric fruits. There was no significant difference in anthocyanin content, fruit quality and size between 500 mg/L ABA treatment and the control group. The effective ABA concentration of increasing anthocyanin accumulation in blueberry is about three times higher than that of grape (*Vitis vinifera* L.) It may be that fruit pruina blocks part of ABA.

For the mechanism by which ABA increases the anthocyanin content, only transcriptome analysis at 24 and 48 h after ABA treatment was carried out (Pilati et al., 2017). It takes approximately 35 days for late green fruits to ripen, and the key genes involved in this process need to be studied in detail. Therefore, we divided blueberry fruits into six stages from late green to mature and analyzed the changes in gene expression throughout the whole process in detail. The transcriptome analysis results showed that the expression of structural genes involved in anthocyanin synthesis was inconsistent. In ABA-treated fruits, the transcripts of CHS, CHI, DFR, and LDOX/ANS, which were proven to be the key structural genes in anthocyanin biosynthesis, were generally expressed at low levels at S1–S2 and at high levels at S5–S6. Previous studies only involved the changes of several structural genes in anthocyanin pathway in mature fruits (Jia et al., 2011; Li et al., 2011). We analyzed the structural gene changes of the whole anthocyanin pathway through transcriptome system to clarify the direct reason for the increase of anthocyanin content during fruit color transformation.

### Potential Key Transcription Factors in Anthocyanin Pathway

Combined with previous studies on transcription factors upstream of these structural genes (Gonzalez et al., 2008), we speculate that the MBW complex is involved in the anthocyanin regulation of the ABA pathway. Homologous genes of MBW transcription factors published in important plants were identified by database annotation and phylogenetic tree construction in blueberry. Two *VcMYBA* (*AtMYB75/90* homologous gene) copies, one *AtMYB56* homologous copy and one *AtGL3* homologous copy were highly correlated with changes in anthocyanin contents. Overexpression of *VcMYBA* (Plunkett et al., 2018) and *AtMYB75/90* (Gonzalez et al., 2008) can enhance anthocyanin synthesis. In addition, *DFR* and *CHS* were also proven to be downstream of *AtMYB75/90*, and *DFR* was downstream of *VcMYBA*. Our results are consistent with those of previous studies. The high expression of *VcMYBA* at S5–S6 was consistent with the change in anthocyanins, and the same structural genes, *DFR* and *CHS*, were highly expressed. *Atmyb56* needs sucrose induction, and its anthocyanin increasing effect is relatively low (Jeong et al., 2018). The homologous gene of *AtMYB56* in blueberry began to be highly expressed at S3, suggesting that this gene may not play a major role. *AtGL3* can enhance downstream anthocyanin expression by interacting with *AtMYB75/90* (Zhang et al., 2003). Therefore, *VcMYBA* may play a key role in ABA pathway.

### Similarities and Differences Between *VcMYBA* and *VvMYBA*

Grape, which is also a non-climacteric fruit similar to blueberry, can increase anthocyanin accumulation under drought (Castellarin et al., 2007), low temperature (Cohen et al., 2012) and ABA treatment (Zhai et al., 2017). *DFR*, *LDOX, UFGT*, and *VvMYBA1* are upregulated under water deficit (Castellarin et al., 2007), and the promoters of both *VvMYBA1-2* and *VvMYBA2* can be activated by ABA, but only *VvMYBA2* can be activated by ethylene (Zhai et al., 2017). White grapes arose through the mutation of *VvMYBA1-2* and *VvMYBA2* (Walker et al., 2007). These findings indicate that *VvMYBA* plays a role in the ABA pathway of non-climacteric fruits and that *DFR*, *LDOX*, and *UFGT* are downstream of *VvMYBA*. For *VcMYBA*, heterologous expression of *VcMYBA* in *Nicotiana benthaminana* could induce anthocyanin accumulation (Plunkett et al., 2018). Through promoter element analysis, there are four promoter elements ABRE responding to ABA in the promoter region of *VcMYBA* (Supplementary Table 5). Transient silencing of the exon region of vcmyba in blueberry fruit significantly reduced the synthesis of anthocyanin in blueberry fruit, while silencing the intron region did not significantly reduce the synthesis of anthocyanin in blueberry fruit. Therefore, it can be considered that the copy of *VcMYBA* that can be translated into protein can effectively regulate anthocyanin synthesis, and whether other copies play a role as lncRNA remains to be further studied.

This study is based on many previous studies, and the results are consistent with the previous conclusions. These results preliminarily explain the mechanism by which ABA increases the anthocyanin content in non-climacteric fruits and broadens the MYB pathway in response to ABA. As ABA is a stress response hormone, these results also provide a reference for explaining the stress-induced increase in the anthocyanin content in plants. This result can provide a basis and new target for anthocyanin breeding in blueberry.

## Conclusion

The application of exogenous ABA in the late green period of blueberry fruit maturation resulted in an increase in anthocyanins. The most direct reason for this result is that *CHS*, *CHI*, *DFR*, and *LDOX/ANS* are highly expressed at S5–S6. Among the transcription factors whose expression changes, *VcMYBA* had the greatest correlation with the changes of anthocyanin and corresponding synthetic structural genes. The promoter of *VcMYBA* has ABRE element, and the up-regulated expression of *VcMYBA* is related to the changes of *ABF* gene family expression, indicating that *VcMYBA* may respond in the ABA pathway. Through VIGS mediated transient silencing, *VcMYBA* has been proved to play an important role in anthocyanin synthesis.

## Data Availability Statement

The original contributions presented in the study are publicly available. This data can be found here: National Center for Biotechnology Information (NCBI) BioProject database under accession number PRJNA664011.

## Author Contributions

TH and WL designed the experiments. TH performed the experiments and analyzed the data. WL and WW wrote the manuscript. All authors read and approved the final manuscript.

## Conflict of Interest

The authors declare that the research was conducted in the absence of any commercial or financial relationships that could be construed as a potential conflict of interest.

## Publisher’s Note

All claims expressed in this article are solely those of the authors and do not necessarily represent those of their affiliated organizations, or those of the publisher, the editors and the reviewers. Any product that may be evaluated in this article, or claim that may be made by its manufacturer, is not guaranteed or endorsed by the publisher.

## Footnotes

1
http://gigadb.org/dataset/100537

2
https://www.uniprot.org/

3
http://www.genome.jp/kegg/

## Supplementary Material

Supplementary Figure 1 Hardness and Brix fruits under ABA treatment. **(A)** The hardness of fruits under 0, 500, and 1,000 mg/L ABA treatment from S4 to S6. **(B)** The Brix of fruits under 0, 500, and 1,000 mg/L ABA treatment from S4 to S6. Different letters indicates statistical significance (*P* < 0.05) as determined by a one-way ANOVA test (Duncan’s multiple range).

Supplementary Figure 2 Identification of known bHLH transcription factors regulating anthocyanin synthesis in blueberry. Phylogenetic tree of bHLH transcription factors was constructed in whole nucleic acid sequences using maximum likelihood tree with 1,000 bootstrap in IQ-tree. The optimal alternative model was selected after calculation.

Supplementary Figure 3 Identification of known WD40 transcription factors regulating anthocyanin synthesis in blueberry. Phylogenetic tree of WD40 transcription factors was constructed in whole nucleic acid sequences using maximum likelihood tree with 1,000 bootstrap in IQ-tree. The optimal alternative model was selected after calculation.

Supplementary Figure 4 Verification of transcriptional results by qRT-PCR. The △△Ct method was applied to each gene pair, and the sample with the highest Ct value smaller than 35 was chosen as the control. Different letters indicate statistical significance (*P* < 0.05) as determined by a one-way ANOVA test.

Supplementary Figure 5 Gene location and sequence analysis of five copies of *VcMYBA*. **(A)** Gene location of five copies of *VcMYBA.* **(B)** Sequence alignment of four copies of *VcMYBA*. The box in the figure shows the code shift area caused by insertion mutation.

Supplementary Figure 6 Heatmaps of the gene expression changes of *ABF*, *SnRK2*, and *PYL* gene families in 1,000 mg/L ABA treatment group compared with the control group during S1–S6. Gene changes were expressed by log_2_ fold change.

Supplementary Table 1 Mean fresh fruit length, diameter and weight throughout ripening. Data are means ± SE (*n* = 10). Different letters indicates statistical significance (*P* < 0.05) as determined by a one-way ANOVA test (Duncan’s multiple range).

Supplementary Table 2 The overall statistical analysis of bases and reads of each samples.

Supplementary Table 3 The overall statistical analysis of reads mapping of each samples.

Supplementary Table 4 Primers used in qRT-PCR.

Supplementary Table 5 Four copies of *VcMYBA* promoter region elements were predicted. The prediction is made by online database PLACE.

Supplementary Table 6 Primers for VIGS, including enzyme digestion sites.

## Abbreviations

CHS: chalcone synthase
CHI: chalcone isomerase
F3H: flavonoid 3′ - hydroxylase
F3′5′H: flavonoid 3′,5′-hydroxylase
DFR: dihydroflavonol 4-reductase
ANS: anthocyanidin synthase
UFGT: flavonoid-3-O-glucosyltransferase.
