# Supplementary figures and images for: Transcriptome Analysis Revealed the Mechanism by Which Exogenous ABA Increases Anthocyanins in Blueberry Fruit During Veraison
Source: Front Plant Sci. 2021 Nov 11;12:758215. doi: 10.3389/fpls.2021.758215 (PMC8632357; doi:10.3389/fpls.2021.758215)

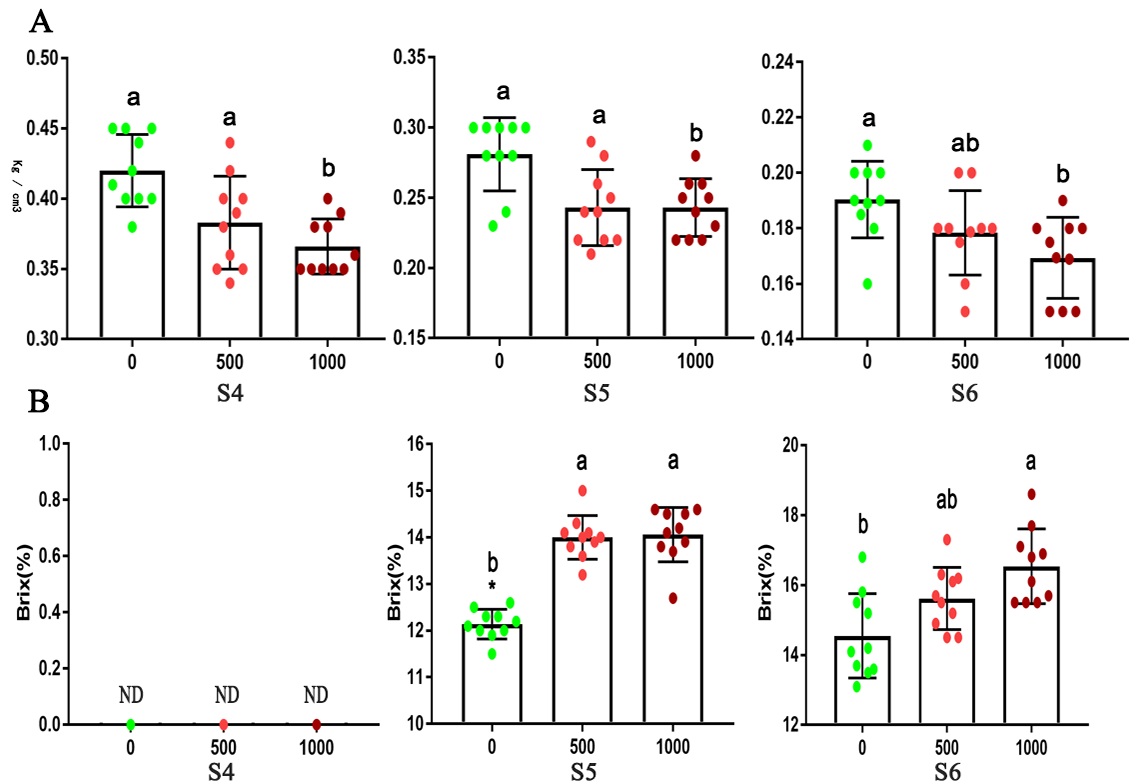

Supplement: Supplementary Figure 1 — Hardness and Brix fruits under ABA treatment. (A) The hardness of fruits under 0, 500, and 1,000 mg/L ABA treatment from S4 to S6. (B) The Brix of fruits under 0, 500, and 1,000 mg/L ABA treatment from S4 to S6. Different letters indicates statistical significance (P < 0.05) as determined by a one-way ANOVA test (Duncan’s multiple range). [file Image_1.TIF]

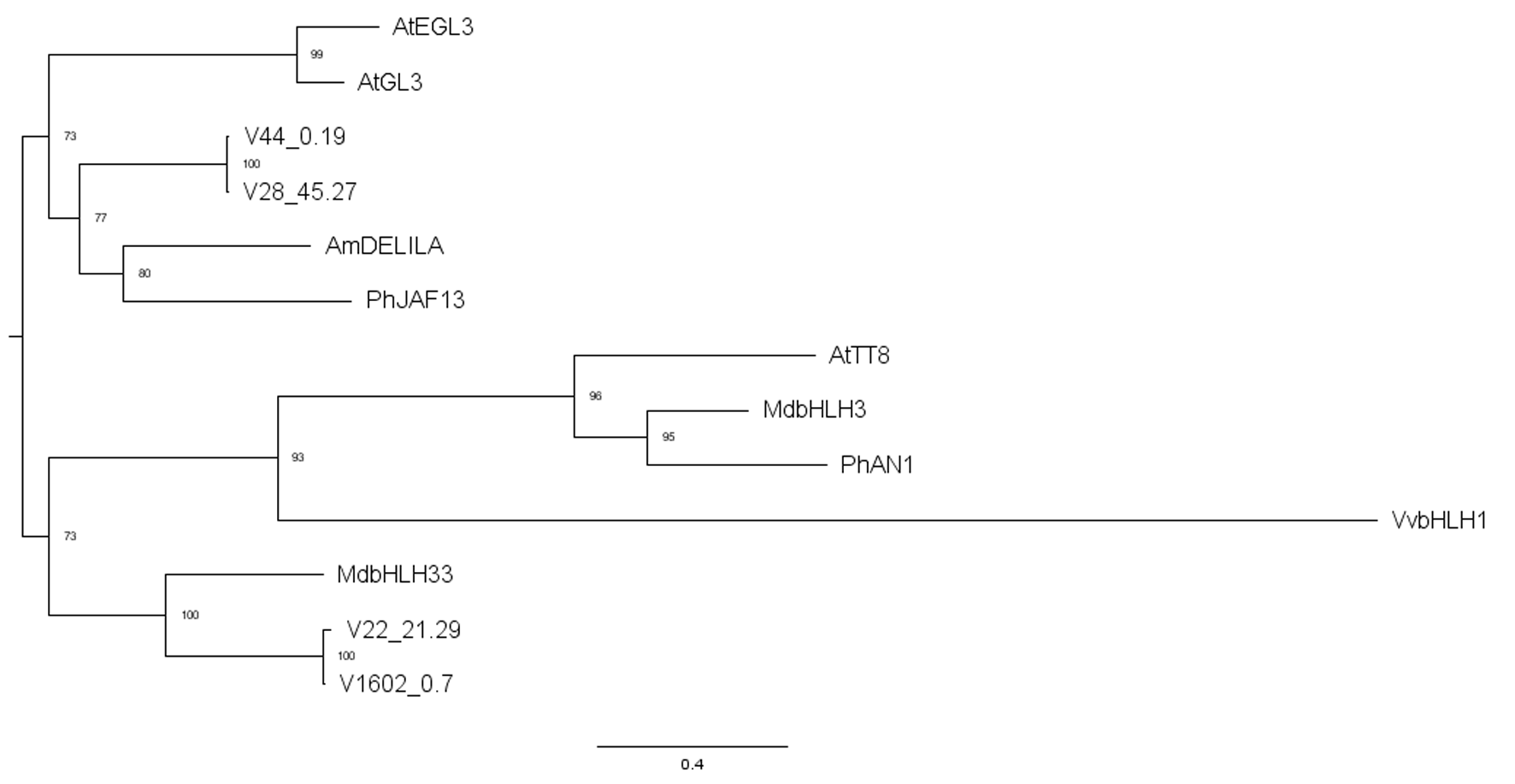

Supplement: Supplementary Figure 2 — Identification of known bHLH transcription factors regulating anthocyanin synthesis in blueberry. Phylogenetic tree of bHLH transcription factors was constructed in whole nucleic acid sequences using maximum likelihood tree with 1,000 bootstrap in IQ-tree. The optimal alternative model was selected after calculation. [file Image_2.PNG]

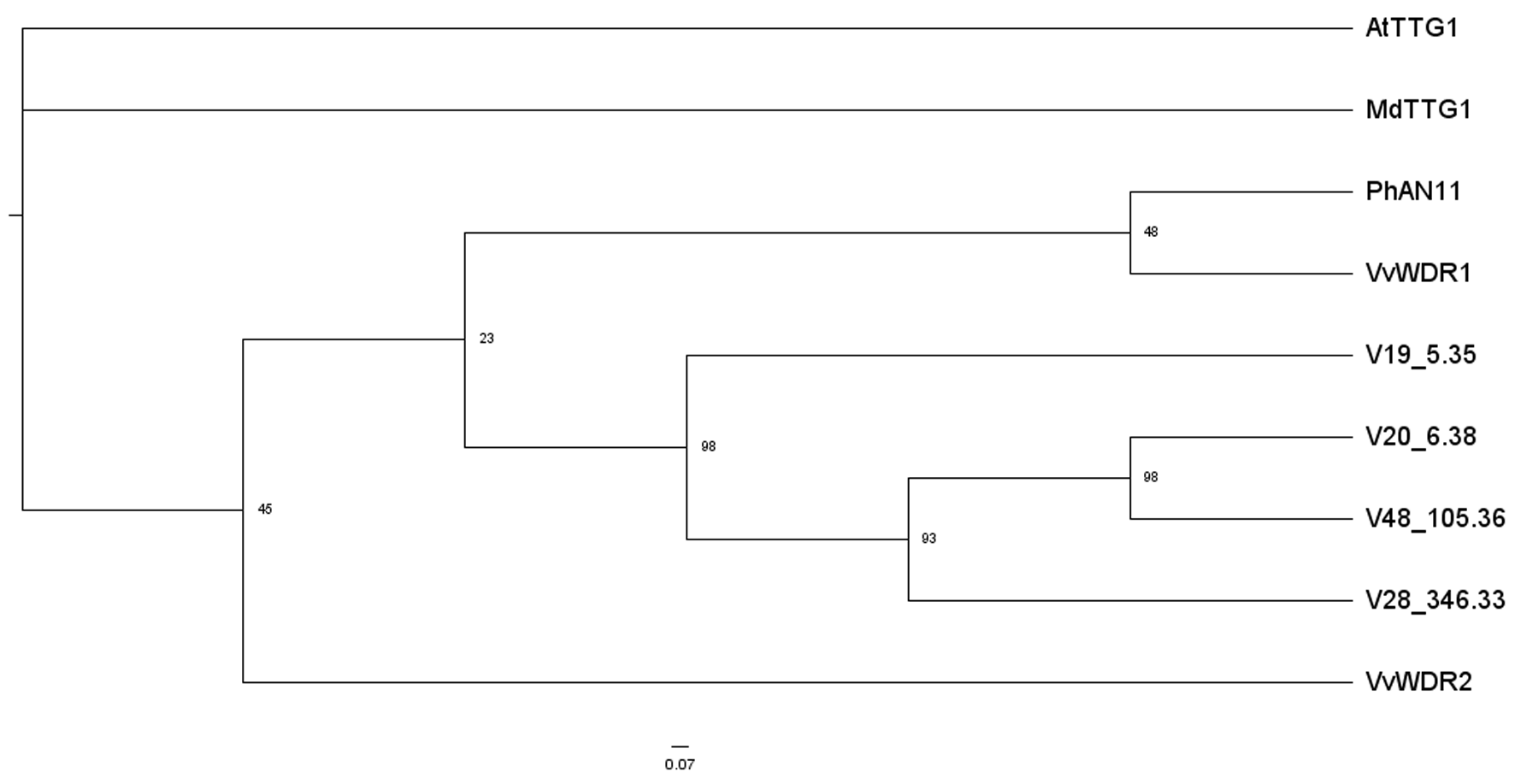

Supplement: Supplementary Figure 3 — Identification of known WD40 transcription factors regulating anthocyanin synthesis in blueberry. Phylogenetic tree of WD40 transcription factors was constructed in whole nucleic acid sequences using maximum likelihood tree with 1,000 bootstrap in IQ-tree. The optimal alternative model was selected after calculation. [file Image_3.PNG]

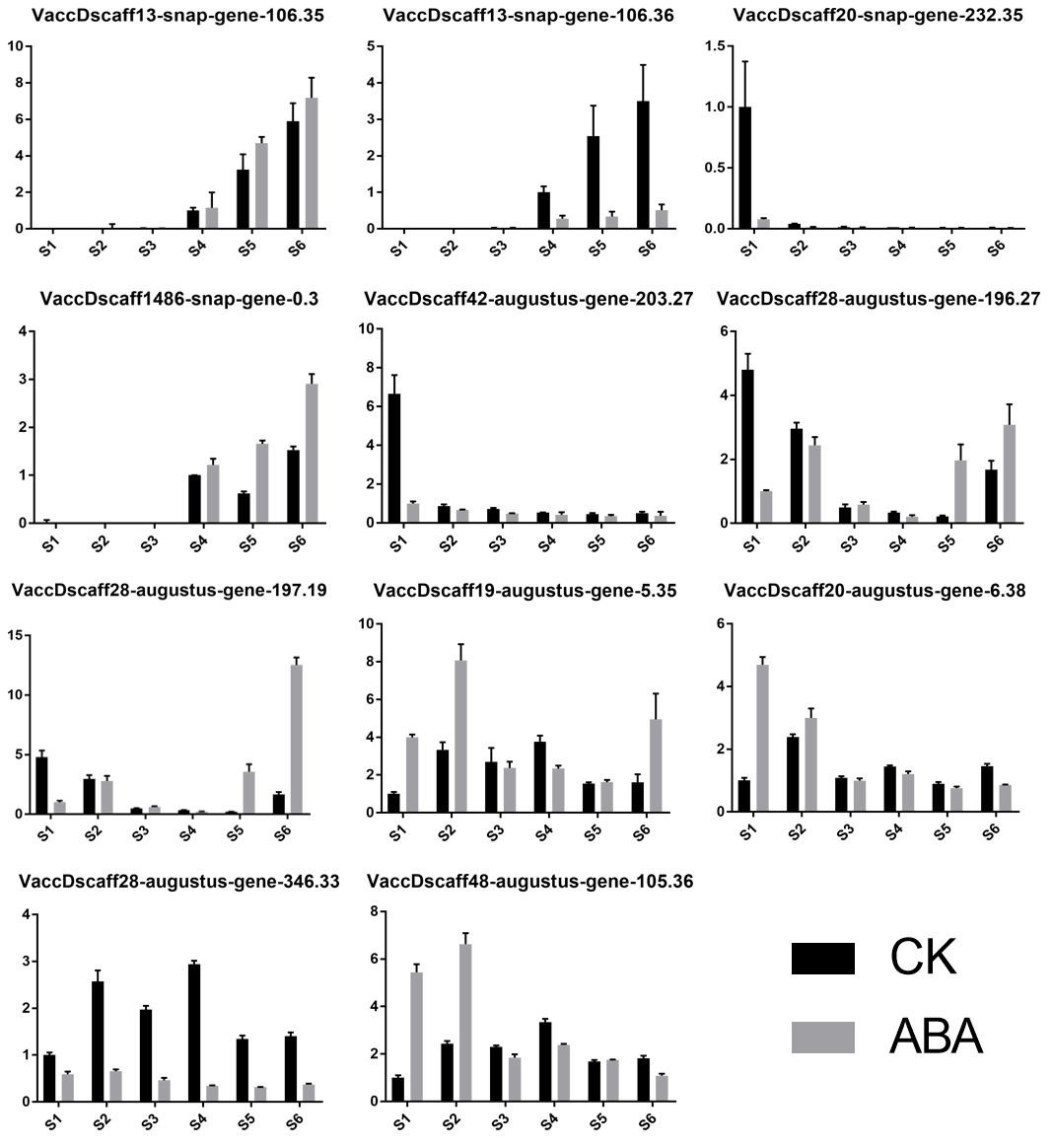

Supplement: Supplementary Figure 4 — Verification of transcriptional results by qRT-PCR. The △△Ct method was applied to each gene pair, and the sample with the highest Ct value smaller than 35 was chosen as the control. Different letters indicate statistical significance (P < 0.05) as determined by a one-way ANOVA test. [file Image_4.TIF]

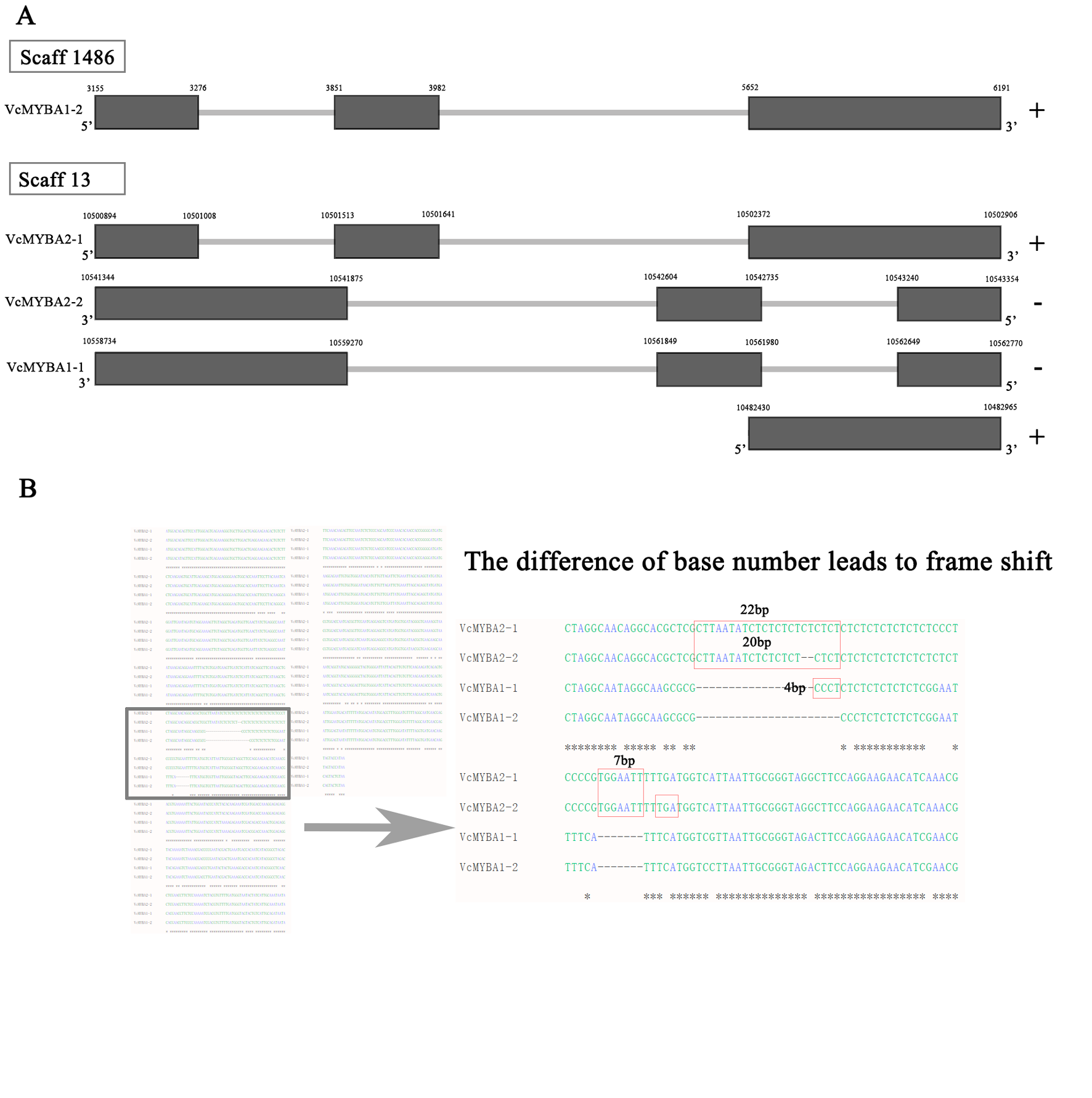

Supplement: Supplementary Figure 5 — Gene location and sequence analysis of five copies of VcMYBA. (A) Gene location of five copies of VcMYBA. (B) Sequence alignment of four copies of VcMYBA. The box in the figure shows the code shift area caused by insertion mutation. [file Image_5.TIF]

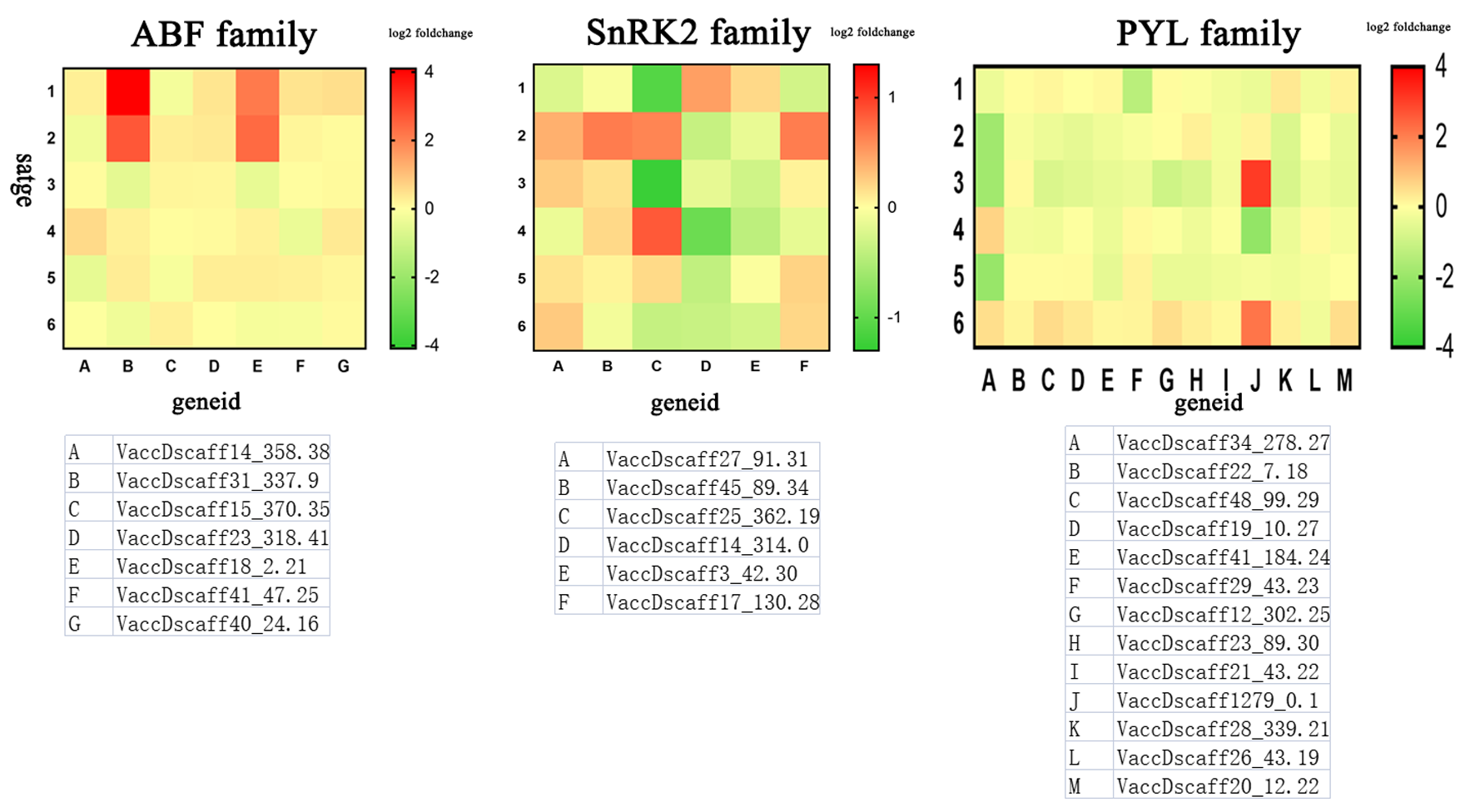

Supplement: Supplementary Figure 6 — Heatmaps of the gene expression changes of ABF, SnRK2, and PYL gene families in 1,000 mg/L ABA treatment group compared with the control group during S1–S6. Gene changes were expressed by log2 fold change. [file Image_6.TIF]
